# Harnessing artificial intelligence for the diagnosis and treatment of neurological emergencies: a comprehensive review of recent advances and future directions

**DOI:** 10.3389/fneur.2024.1485799

**Published:** 2024-10-11

**Authors:** Majd A. AbuAlrob, Boulenouar Mesraoua

**Affiliations:** ^1^Department of Neurosciences, Hamad Medical Corporation, Doha, Qatar; ^2^Weill Cornell Medical College, Doha, Qatar

**Keywords:** artificial intelligence, AI in diagnosis, neurology, neurology emergencies, AI in epilepsy

## Abstract

Artificial intelligence (AI) is rapidly transforming the landscape of neurology, offering innovative solutions for diagnosing and managing emergent neurological conditions such as stroke, traumatic brain injury, and acute spinal cord injury. This review critically examines the recent advancements in AI applications within the field of neurology, emphasizing both the potential and limitations of these technologies. While AI demonstrates remarkable accuracy and speed in diagnostic imaging, outcome prediction, and personalized treatment plans, its integration into clinical practice remains challenged by ethical concerns, infrastructural limitations, and the “black box” nature of many AI algorithms. The review highlights the current gaps in literature, particularly the limited research on AI’s use in low-resource settings and its generalizability across diverse populations. Moreover, the review underscores the need for more longitudinal studies to assess the long-term efficacy of AI-driven interventions and calls for greater transparency in AI systems to enhance trust among clinicians. Future directions for AI in neurology emphasize the importance of interdisciplinary collaboration, regulatory oversight, and the development of equitable AI models that can benefit all patient populations. This review provides a balanced and comprehensive overview of AI’s role in neurology, offering insights into both the opportunities and challenges that lie ahead.

## 1 Introduction

Background the rapid advancement of artificial intelligence (AI) has brought revolutionary changes in major healthcare sectors, with improved tools and innovative ways for diagnosis and patient management. AI has shown a lot of promise in the field of neurology in the present day as regards both complex neurological disorder diagnosis and treatment. For instance, healthcare providers are tremendously challenged by neurological disorders due to stroke, traumatic brain injuries, epilepsy, among other acute situations, because of their complex nature and timely intervention needed in these cases. This is even more critical in emergent cases, where rapid and accurate diagnosis is needed for the right intervention on a patient. The application of AI in neurology is an extrapolation of successes made in other general areas of medicine, where large datasets have been analyzed by machine learning algorithms, neural networks, and other AI technologies to help predict outcomes and guide clinical decision-making. AI’s ability to process vast volumes of information quickly and accurately makes it a potent tool in the emerging diagnosis and treatment of neurological disorders, where time plays a critical role (1).

Problem Statement Although there have been enormous advancements in medical imaging, clinical protocols, and pharmacological treatments, timely diagnosis and effective treatment of emergent neurological conditions remains a herculean task. Also, traditional diagnostic methods are usually very time-consuming and require a lot of expertise, such as experience and, for the said reason, may not be easily accessed in case of an emergency (44). Similarly, complex neurological disorder treatments need to be customized for individual patients, making standard approaches less effective. AI has in it the potential to bridge these gaps with real-time diagnostic support and customizing treatment plans, based on huge datasets and predictive modeling. While this is its potential, AI is only at the very early stages of actual practice applications, such as emergent neurological cases. Current advancements are in need of comprehensive scrutiny due to the state of the field in the diagnosis and treatment of neurological disorders with AI, which suggests specific places for further method development and research.

While the advancements in AI for neurological care are promising, it is critical to acknowledge gaps in the current literature. Many existing reviews provide a broad overview of AI applications but fail to focus on emergent neurological situations where time-sensitive interventions are paramount. This article aims to fill that gap by reviewing the specific role of AI in diagnosing and managing acute neurological emergencies such as stroke, traumatic brain injury, and acute spinal cord injury. Moreover, prior reviews have not sufficiently discussed the ethical and practical challenges of integrating AI into real-world clinical settings, especially in low-resource environments. This comprehensive review will address these overlooked areas, providing a nuanced perspective on future research directions.

Aims the primary intention of the literature review is to assess the current state of AI-assisted diagnosis and treatment of neurological disorders, with a particular focus on emergent cases. This review shall hence strive to:

- Discuss recent developments in AI technologies relevant to emergent neurological disease diagnosis and treatment.
- Evaluate how well AI applications improve patient outcomes in the emergency setting.
- Highlight challenges and limitations in the implementation of AI in neurology.
- Make recommendations for future research and clinical practice.

A well-structured review of this topic is essential to facilitate a clear understanding of AI’s transformative potential in neurology. This review will follow a systematic format to ensure coherence and clarity, covering recent technological developments, clinical applications, and the ethical implications of using AI in emergent neurological cases. By structuring the discussion around specific neurological emergencies, this review avoids the pitfalls of other articles that lack focus and cohesion. Additionally, we aim to highlight the limitations of current AI applications, thereby setting the stage for targeted research that can address these shortcomings in a meaningful way.

Purpose and Usefulness As previously outlined, this literature review will cover studies published in the last 8 years that echo the most recent advances in AI and its applications in neurology. This review, therefore, looks at AI tools aiding in diagnoses, treatment planning, and management strategies for dealing with emergent cases of neurological disorders. Since there is an increased need for timely and accurate interventions in emergent cases, this review will focus on the role of AI on patient outcomes during such incidences (2).

## 2 Methodology

This literature review was conducted to critically assess the role of artificial intelligence (AI) in the diagnosis and treatment of neurological emergencies, with a particular focus on emergent neurological conditions such as stroke, traumatic brain injury (TBI), and acute spinal cord injury. The review was structured to identify recent advancements, challenges, and future directions in AI applications within the neurology field.

### 2.1 Literature search strategy

The literature search was performed using several academic databases, including PubMed, IEEE Xplore, Google Scholar, and Scopus, to identify peer-reviewed articles published between 2015 and 2023. The following key search terms were used, either individually or in combination:

- “Artificial intelligence”
- “Neurology”
- “Emergent neurological conditions”
- “Stroke diagnosis”
- “Traumatic brain injury”
- “Machine learning in healthcare”
- “Deep learning in neurology”
- “AI in medical imaging”
- “AI in EEG analysis”
- “AI in neurogenomics”
- “Cognitive disorders and AI”

Articles that specifically addressed the use of AI for diagnosis, treatment planning, and outcome prediction in neurological conditions were included. Both retrospective and prospective studies, case studies, systematic reviews, and meta-analyses were considered.

### 2.2 Inclusion and exclusion criteria

The review included studies based on the following criteria:

**Inclusion Criteria:**

- Published in English.
- Focused on AI applications in neurology or healthcare.
- Provided empirical evidence of AI’s performance in diagnosing, treating, or managing neurological emergencies.
- Studies involving AI’s ethical and practical challenges in real-world clinical settings.

**Exclusion Criteria:**

- Studies that primarily focused on non-neurological conditions.
- Articles with limited access to full texts.
- Studies published before 2015 (except landmark studies that contributed foundational knowledge to the field).
- Non-peer-reviewed articles, abstracts, and commentaries.

### 2.3 Data extraction and analysis

Relevant data from the selected studies were extracted into a standardized table. This table included key information such as the type of AI model used (e.g., machine learning, deep learning), the neurological condition targeted, study outcomes, and the challenges or limitations identified in the study. Each article was reviewed for the AI model’s clinical utility, accuracy, speed, and the ability to integrate with traditional healthcare practices.

### 2.4 Synthesis of findings

The findings from the reviewed studies were synthesized to identify patterns in the successful application of AI technologies in neurology. Special attention was given to AI’s role in emergent neurological conditions where timely diagnosis and intervention are critical. The review also synthesized information on the barriers to AI implementation, including ethical considerations, data privacy concerns, biases in AI models, and the integration of AI in low-resource settings. Challenges such as the “black box” nature of AI and the difficulty in clinical validation were also discussed based on the literature reviewed.

### 2.5 Quality assessment

To ensure the robustness of the findings, each study was critically evaluated based on its methodology, sample size, and the rigor of its AI model validation. Biases and limitations of each study were documented to provide a comprehensive and balanced view of AI’s potential in neurology.

### 2.6 Ethical considerations

Ethical implications were also extracted and analyzed, particularly concerning data privacy, bias in AI models, and the need for transparency and explainability in AI-driven clinical decision-making. Studies that explored the ethical dimensions of AI in healthcare were incorporated to highlight gaps and provide recommendations for future research and clinical practice.

## 3 AI in healthcare: an overview

General Applications of AI in Healthcare: Artificial intelligence very much remains an integral part of the modern-day healthcare paradigm, ranging from predictive analytics to personalized medicine. Fundamentally, AI utilizes machine learning algorithms, neural networks, and natural language processing for the analysis of huge data sets to identify patterns and make predictions. AI, thus empowered, has helped improve the very core elements of healthcare: diagnosis, treatments, planning, drug discovery, and patient management.

A good application for this is diagnostic imaging, in which AI algorithms are being trained to analyze medical images like X-rays, MRIs, and CT scans with high accuracy. The studies have shown that AI systems could be comparable or even better than human experts when finding medical image abnormalities, thus making correct and faster diagnoses (3). For instance, in oncology cases where AI was applied in the detection of cancerous lesions on radiological images, a lot of time gets saved during detection, with a result of better patient outcomes (4).

Besides imaging, AI is also applicable in predictive analytics for the identification of patients at risk of developing some diseases. Artificial intelligence using electronic health records and other clinical data provides a predictive way of the probability of an occurrence of a disease, hence enabling intervention measures to be instituted even for preventive purposes (5). This is of great use in the prognosis of chronic illnesses such as diabetes and cardiovascular diseases, whereby complications and adverse effects are prevented in order to reduce healthcare expenses.

### 3.1 AI in neurology

The field of neurology has advanced greatly through the application of AI to combat the complexities associated with neurological disorders. Symptoms of neurological conditions are generally hard to quantify and follow by traditional techniques, and this is the key argument by proponents of AI such as Rao et al. (6). With its ability to handle huge amounts of data and detect subtle patterns, AI offers a totally new approach toward these diseases.

The application of AI in neurology is best illustrated in diagnostic imaging. The brain images are subjected to analysis of late by use of techniques that include deep learning, convolutional neural networks (CNNs), to detect and diagnose the symptoms of any upcoming potential neurological disorder. For example, AI algorithms used to find early signs of Alzheimer’s disease by studying the structure and functional MRI scans can detect those even before the clinical symptoms start to manifest in patients (7). This helps in early detection, which could play a very important role in slowing the advance of the disease and improving the quality of life for such patients.

Besides imaging, the application of AI is seen in the analysis of EEGs for various epileptic seizure disorders and other such disorders. An analysis of an EEG by conventional procedures is both time-consuming and needs expert interpretation. AI-based systems can automate the procedure and thereby result in a quick and at-the-same-time accurate diagnosis (8). This is particularly relevant in the emergent cases of neurology, where timely diagnosis can go a long way in determining outcome post-therapy.

Emerging Technologies in AI-Assisted Neurology: Currently, several emerging technologies are underway and will shape AI research in neurology with the potential to revolutionize diagnosis and treatment. For example, one of these technologies would be deep learning, a distinctive way of modeling complicated patterns within the data. In recent years, deep learning algorithms have been utilized to comprehend various neurological conditions’ disease mechanisms and possible therapeutic targets, which seemed to increase in relevance considerably recently (9).

Reinforcement learning, a subfield of machine learning, is another promising technology where algorithms learn through the environment they interact with to make decisions. In neurology, reinforcement learning has been applied in the development of neurorehabilitation systems during interaction with patients—a characteristic AI device that caters to changes in progression, offering personalized therapy options (10).

In addition, NLP is increasingly being applied in neurology for the purposes of the analysis of patient records and clinical notes. Valuable information, especially on symptoms, treatment responses, and disease progression, present in free text, can be obtained from the application of NLP algorithms in regard to a patient’s condition (11).

### 3.2 AI in emergent neurological cases

Emergent neurological cases, such as stroke, traumatic brain injury, and acute spinal cord injury, demand timely management. AI has proved its potential in such cases by giving rapid diagnostic support and aiding at critical junctures of decision-making (12). For example, AI algorithms were created in stroke cases to analyze imaging data and identify patients who would benefit from thrombolysis or thrombectomy; this vastly reduced the time required for treatment and resulted in better outcomes.

The use of AI systems to predict outcomes and guide decisions on treatment with data from the patient, including imaging, vital signs, and clinical history, is tremendous. Such AI-enabled insights would aid clinicians in making prioritized interventions and better allocate resources in settings with constrained resources.

### 3.3 Limitations of AI in neurological emergencies

While the potential for AI in neurology, particularly in emergent situations, is well-documented, the technology is not without limitations. One major challenge is the “black box” nature of many AI algorithms, especially those based on deep learning models. These models can generate accurate predictions, but their inner workings are often opaque to clinicians, leading to concerns about the interpretability of AI-assisted diagnoses and treatments (13). This lack of transparency can hinder trust and adoption in clinical settings, as healthcare providers may be hesitant to rely on systems they do not fully understand.

Moreover, AI algorithms are only as good as the data they are trained on. Bias in AI systems is a well-recognized issue, particularly in healthcare, where training data may not adequately represent diverse patient populations. For example, AI systems trained predominantly on data from Western populations may not perform as well for patients from different ethnic backgrounds, potentially leading to misdiagnoses or suboptimal care (14). This raises ethical concerns, especially in high-stakes emergent cases where AI recommendations could significantly influence patient outcomes.

Furthermore, the clinical validation of AI tools in real-world settings remains a significant hurdle. Many AI systems have shown promising results in controlled research environments but have yet to be thoroughly tested in everyday clinical practice, particularly in low-resource settings. The transferability of these technologies to varied healthcare environments is still questionable, as resource limitations may affect the deployment and reliability of AI systems (15). These limitations underscore the need for more comprehensive and transparent AI systems that can be trusted by clinicians and patients alike.

## 4 Computer-aided diagnosis of neurological disorders

### 4.1 AI in diagnostic imaging

One of the most important fields in which AI has contributed much to Neurology is Diagnostic Imaging. Traditional imaging techniques—beans, CT scans, and PET scans—are basically diagnostic pieces of equipment for neurological disorders. Still, their interpretation may be pretty subjective and time-consuming, needing highly specialized radiologists. AI has emerged as a power tool in the realm of imaging analysis, especially for improving accuracy and efficiency through deep learning algorithms.

Even though the recent research evidence has been able to illustrate that AI can be applied in neurological conditions diagnosis using imaging, the history of artificial intelligence implementation dates back to over three decades ago. For instance, CNNs were designedly trained for the detecting early signs of Alzheimer’s disease by reviewing structural MRI scans. The AI systems showed an accuracy close to that of expert radiologists with faster processing times added as an advantage (4). Early diagnosis of Alzheimer’s disease is important for timely institution of intervention measures that might retard the process.

AI has already been used in providing great promise to assist in a rapid diagnosis concerning emergent neurology cases, such as acute ischemic stroke. Stroke is a significant cause of worldwide disability and death with a narrow window for effective treatment. Algorithms have been developed that are AI-driven for image analysis in CT and MRI, which one can leverage quickly to capture stroke type and occluded blood vessels and assess the extent of damage in the brain. Diagnostic tools like these, powered by AI-driven innovation, would increasingly help reduce time to treatment, a critical determinant of patient outcome (16).

### 4.2 AI in EEG analysis

Electroencephalography is another vital tool in establishing the diagnosis of neurological disorders, more especially in epilepsy. Traditionally, the analysis of EEG data used to take a lot of time since it required manual interpretation by neurologists. AI has revolutionized EEG analysis with automatization of detecting abnormal brain wave activity associated with seizures and other neurological conditions.

Deep learning models, particularly recurrent neural networks and long short-term memory networks, have been applied to EEG data in epileptic seizure prediction with a high degree of accuracy. The models are capable of going through a large data set from EEG recordings to come up with a pattern indicating seizure events that may not be observable by human eyes at any immediate instance. This is particularly useful in view of emergency situations where the time factor is pretty crucial for diagnosis and intervention.

Nevertheless, application of AI to seizure prediction has opened a window for preventive interventions. According to some studies, AI models trained with EEG data predict seizures minutes or even hours in advance, thus offering sufficiently large benefits for epilepsy management.

### 4.3 AI in the diagnosis of traumatic brain injury (TBI)

TBI is one of those critically important conditions that require immediate attention and treatment to prevent long-term neurological damage or even death. Diagnosis in cases of TBI mostly involves the use of imaging techniques, particularly CT, to determine the extent of injury to the brain. Traditional imaging techniques have usually failed in detecting subtle changes to brain structure and function following TBI due to its subtle nature of alterations.

More recently, AI has been, this way, applied to support the diagnosis of TBI via analysis of imaging data, with clinical variables down the line. Machine learning models have been constructed to better predict the outcomes for TBI patients from CT scan data, vital signs, and others like clinical indicators. AI-driven predictions could input into critical decisions about appropriateness related to the urgency of intervention, the need for ICU care, and potential for recovery (17).

AI applications in neuropsychological assessments used for the diagnosis of TBI have also been applied alongside imaging. For example, the speech and language patterns of TBI patients were examined to glean insights into such cognitive impairments that lie hidden in imaging alone using NLP algorithms. It is then that this kind of multi-modal approach to diagnosis, combining imaging, clinical data, and neuropsychological assessment, held great promise in management with regard to TBI.

### 4.4 Case studies and applications to real life

Several case studies and real-world applications have attested to the effectiveness of AI in diagnose neurological disorders. For example, McKinney et al. (18) conducted a study that revealed the use of AI in the diagnosis of an acute stroke in a large clinical setting. It reported an AI system that could identify stroke on computed tomography scanner images virtually as well as expert radiologists do—yet significantly eased diagnosis time. This time-to-diagnosis reduction is incisive in emergent cases where every minute counts.

Another very good example is the application of AI in Parkinson’s disease diagnosis. AI models are trained using MRI and PET scans to detect the first manifestations of Parkinson’s, often before motor symptoms start to set in. Early diagnosis instituting treatments that slow down the progression of the disease drives better patient outcomes.

AI has been successfully applied to epilepsy care in recent times, in relation to patient monitoring and seizure prediction. For instance, Kuhlmann et al. (19) presented an example where AI was applied in predicting seizures for patients with refractory epilepsy. The system supplied proper predictions of seizure episodes, facilitating interventions on time and thus reducing the frequency of seizures.

Case examples in the next sections demonstrate the potential for AI to greatly affect a sea-change in neurological disorder diagnosis. As the development of AI progresses, so will the applications move into neurology, opening more opportunities in routine and emergent cases for early and accurate diagnoses.

## 5 AI in treatment and management of neurological disorders

### 5.1 AI-guided treatment plans

Especially with neurology, where patients require personalized treatment approaches because of the nature of neurological disorders—both complicated and variable—AI in treatment planning has shown remarkable promise. Recent AI-driven systems analyze big datasets, like patient history, genetic information, and clinical biomarkers, in developing personalized treatments that fit the patients’ profile. These include state-of-the-art innovations in treatment planning for patients with epilepsy. This is considered important in such conditions as epilepsy, multiple sclerosis, and Parkinson’s, for the treatment response of which patients sometimes show a widely differing extent.

However, AI has been used to build models informed by a lot of data obtained through clinical trials and the outcomes of real-world cases related to predicting the optimal medication dosage for patients with epilepsy in a way that minimized risks yet maximized therapeutic efficacy (20). The models use machine learning algorithms to study data obtained from analyses of clinical trials and real-world patient outcomes, hence giving out better treatment options.

In the field of Parkinson’s disease management, AI has been utilized in the optimization of deep brain stimulation (DBS) therapy processes. DBS is a well-known and effective treatment for Parkinson’s, but its effectiveness depends on the precision of electrode placement and the setting of parameters, which are difficult to optimize by hand. Unfortunately, the management of systems with more complex representation is not that easy and could be done manually. AI-based algorithms to analyze patient-specific information reportedly lead to optimal DBS parameters, new treatment outcomes, and improvements. A good overview of the field was discussed and summarized by Kühn and Volkmann (21).

### 5.2 Surgical assistance

Neurosurgery is one of the tough-to-handle surgical specialties and needs relatively greater precision and technique. With this regard, AI has started to implement neurosurgical procedures, aiming to deliver real-time decision support and improve the surgical outcome produced in return. AI-driven surgical robotics and navigation systems have been reported in neurological surgeries with elements of complexity, such as resection of tumors and interventions to address spinal surgery (22), in line with that.

A significant number of applications for AI implementation in neurosurgery relate to brain tumor surgical planning and execution. Conventional surgical planning depends on the surgeon’s experience and judgment, although AI systems are capable of conducting preoperative imaging data analysis for creating 3D models, hence the neuronavigation tackle relevant to information on these models. Such showcases work to let surgeons know exactly where to make the incision so that they cause as little damage as possible to the healthy tissue around a tumor (23).

In this sense, an AI-powered robotic system can take part in helping the instruments get feedback during a surgery, maintaining its steadiness and offering real-time feedback to the surgeon. With this approach, it will be more accurate, will reduce the effect of human fallibility, and will allow undertakings that are less aggressive. For example, AI-supported robotic systems, such as ROSA and Mazor X, have led to increased surgical accuracy and decreased recovery times in spinal surgeries and deep brain stimulation procedures, respectively. AiBootApplication.

### 5.3 Rehabilitation and long-term management

Notably, the role of AI applies beyond acute treatment to rehabilitation and long-term management in neurological disorders. Motor and cognitive recovery in patients suffering from rehabilitation period.

TrueAIhas helped develop the leading personal rehabilitation programs, which are based on patients’ progress. Machine learning algorithms analyze rehabilitation session data to read patterns of movement and recovery metrics, in order to make real-time adjustments to therapy protocols. For example, AI-enabled exoskeletons and robotic devices provide assistance to patients in carrying out repetitive movements involved in physical therapy, with features like real-time feedback and setting resistance according to the ability of the patient. For interventions on a more precise level, robotic devices may sometimes be incorporated into traditional physical therapy. For instance, robotic gait trainers can enable walking movements for (39).

Apart from physical rehabilitation, AI has also been incorporated into cognitive rehabilitation for patients with brain injuries and neurodegenerative diseases. Artificial intelligence-powered platforms can deliver personalized cognitive exercises for the irregularities of a specific patient, can systematically track the progress of the activities, and even modulate the difficulties during the course of the improvement tempo (24). These types of systems can find effective use in further elicitation of cognitive recovery in stroke patients and those suffering from mild cognitive impairment (MCI) (25, 40).

More importantly, AI applications will be important in the long-term monitoring of a patient and management. For instance, wearable devices and mobile health apps with AI abilities will be useful in monitoring vital signs and patterns of movement and symptoms delivery in real time to healthcare providers (26). For instance, AI-driven apps have been useful to monitor the symptoms of Parkinson’s disease like tremors and abnormalities in the gait, allowing the remote adjustment in clinical management for patients with the disease carved out herein (27). Continuous monitoring comes very handy in chronic neurological conditions because it can help reveal the course of chronologically developing symptoms that might prevent later complications and hospitalizations (42).

### 5.4 Case-based studies and real-life applications

Several real-world applications highlight the transformation that the AI field is having in the treatment and management of neurological disorders. A good example is the optimization of the treatment plan for conditions such as strokes. A study by McKinney et al. (18) has also proven that the AI system can review patient information, imaging, and clinical history to give a recommendation for the best thrombolytic therapy or the best surgical operation. This AI-driven approach reduced time to treatment and improved patient outcomes in acute cases of stroke.

Robotic systems incorporated with the help of AI in neurosurgery have shown that successfully conducted complex resections. A case report documented by Shen et al. (22) was performed on the use of an AI-driven robotic system during a resection of glioblastoma, one of the most aggressive forms of brain cancer. The robotic system is capable of providing real-time feedback before and during surgery to allow the surgeon to conduct better resection with minimed damage to neighborhood tissues for good patient outcomes.

AI-exoskeletons have also been used for stroke patients. A study by Mehrholz et al. (28) has shown that stroke patients using AI exoskeletons for their rehabilitation had significantly better mobility and functional independence as compared to those treated with conventional therapy.

These are illustrative of the potential of AI in changing the very landscape of treatment and management in neurological disorders and thus give fresh hope both to the patient and the clinician.

## 6 Ethical and practical concerns

### 6.1 Ethical implications

The excessively rapid uptake of AI in neurological diagnosis and treatment raises numerous very serious ethical issues and, if not handled properly, it could tilt the scales toward responsible and fair use. One major issue of AI that comes to the focus with such a rapid uptake is bias within the algorithms. AI systems in nature are based on gigantic datasets, and lack of representation of diversified population in these datasets may be a significant problem where the developed algorithms continue their existence discretely. For example, research indicates that models of AI that are largely trained on data from certain ethnic groups are less accurate for patients from other backgrounds, with possible outcomes of misdiagnosis—and underdiagnosis—and less than optimal care (14).

Selection of such datasets must be diverse and represent the population to avoid this issue. This also requires that there be transparency in both the development and the deployment stages of the AIs; both the clinicians and patients need to know how the decisions are being made by an AI algorithm and therefore know what limitations or bias are possible (29). Maintaining ethical AI practice also needs ongoing monitoring and validation of the AI systems to prove that they will perform equally well with all patients (41).

Another important ethical issue surrounding AI in healthcare is privacy and data security. The accuracies of AI systems for treating patients come from its training with very large pools of patient data that are essentially medical records, imaging data, and genetic information. Such a sensitive use of data raises concerns about its misuse and unauthorized access or potential breach with such large pools of sensitive personal health information. Privacy and security of patient data are very critical; the data should, therefore, be well protected with strong protection mechanisms to ensure that the data is secure.

Another critical ethical consideration is that of informed consent in using AI in healthcare. It is important that patients are properly informed on how AI systems would be used and the basis of the data to be collected and the use to which such data will be put. Correctly, patients should be given the chance not to receive AI-driven interventions if they would prefer the traditional method of care. Ensuring that patients are aware of their rights and that they have autonomy in their healthcare decisions is essential in maintaining trust in AI technologies.

There comes the issue of accountability and responsibility whenever AI is utilized toward care. Sure, AI systems generate enormous insights and in many instances give recommendations, but in the end, the liability associated with clinical decisions lies with human providers. Of the greatest significance is the guidance on how the recommendations made by AI should be integrated into clinical practice: the final decision regarding the treatment has to rely on the judgment of professionals and patient preferences (30).

Ethical considerations are crucial when discussing the deployment of AI in neurological emergencies. A significant concern is the transparency of AI decision-making processes, often referred to as the “black box” problem. Clinicians and patients alike may struggle to trust AI-driven recommendations without a clear understanding of how the system arrives at these conclusions. Furthermore, regulatory frameworks for AI in healthcare remain underdeveloped, making it challenging to ensure that AI systems meet the required standards of safety and efficacy. Comprehensive guidelines and strict regulatory oversight are needed to prevent misuse and ensure that AI technologies are deployed responsibly.

### 6.2 Practical challenges

Laying aside ethical concerns, some practical ones in respect to how AI can really be transformed and integrated with usable healthcare clinical practices points to problems in regard to interoperability: In this line, most of the hospitals and clinics currently in play lack proper interoperation of sophisticated AI systems and legacy systems of healthcare infrastructure. Serious efforts should be applied to establish appropriate compatibility of the advanced modern AI technologies with the popular legacy systems prevalent in most of the hospitals and clinics. Upgrading these systems to AI tool accommodation would be time and resource-consuming, which will thus represent a critical challenge in making the technologies widely accepted.

In addition, clinical applications will call for significant training of the healthcare workforce. Ideally, the medical workforce should be trained in the utilization of such AI tools, interpret results and integrate them for better understanding of patient care (31). That is bound to call for realignment in the medical-education framework and shift in training programs to allow for the next generation of healthcare associates to be conversant with these AI technologies.

Yet another practical challenge lies in health care AI regulation. The regulatory entities have yet established frameworks for the specific evaluation of safety and efficacy for medical devices and software driven by AI, according to Bakker et al. (32). Assurance that AI systems meet requirements can be a long and painstaking procedure; thus, critical technologies are likely to remain partners in some cases without deployment in clinical settings.

Besides that, the implementation of AI may be very costly, enough for most healthcare institutions, especially the low-resource ones, to not afford. While AI has the potential to cut health service costs in the long term, since it is efficient and enhances health outcomes, the high magnitude of the required investment in infrastructure, training, and integration of AI services could be substantial. Rising to all the financial challenges will be crucial to realize the benefits of AI for all patients regardless of geography or economic status.

Finally, there is the issue of acceptance. Apart from the anticipated improvement that may come from AI, any form of impact or changes in the tasks from work might be among those reasons that the healthcare professional is apprehensive of using the technology because it means dispossession of work, loss of control, and others (15). Embracing AI in neurology as a valuable tool throughout the field, and even beyond it, will be of paramount importance in building trust for these systems and showcasing their clinical validation and value throughout real-world success stories.

### 6.3 Balancing innovation with responsibility

The practical and ethical challenges associated with AI in neurology underline the need for a balanced approach that holds concerns of innovation and responsibility with equal respect. While AI offers the potential to completely revolutionize the process of diagnosis and treatment of neurological disorders, it is, at the same time, very important that development and deployment of such technologies be carried out in a fair manner that is transparent and according to the highest standards of patient care.

This will likely emanate from collaborative working of AI developers, healthcare providers, patients, and regulatory bodies, who might work together in order to ensure AI technologies work in a manner that supports all patients, addresses all ethical concerns, and overcomes all practical challenges. In this way, all potential ways of AI application in neurology would be fully realized, thereby offering new hope for patients with neurological disorders but at the same time maintaining the trust and confidence of the healthcare community.

## 7 Broader applications of AI in neurology

To provide a comprehensive review of AI’s role in neurology, it is crucial to broaden the scope beyond its well-established applications, such as in stroke, Alzheimer’s disease, and epilepsy. AI technologies hold vast potential for addressing a wider range of neurological conditions, particularly those that have historically been underrepresented in the literature. Expanding research in this area would allow AI to demonstrate its versatility and impact across various complex and rare neurological disorders.

One of the emerging areas where AI can play a transformative role is in neurogenomics. AI has the ability to analyze large volumes of genetic data to identify patterns and genetic mutations associated with neurological conditions. For example, AI-driven tools are increasingly being used to predict disease onset and progression in disorders like amyotrophic lateral sclerosis (ALS) and Huntington’s disease (9). By integrating neuroimaging, genetic data, and clinical biomarkers, AI can help clinicians develop personalized treatment strategies tailored to a patient’s unique genetic profile, which is particularly relevant in managing these neurodegenerative diseases where early detection is critical (7).

In addition, AI has shown promise in the management of movement disorders such as Parkinson’s disease. Beyond diagnostic imaging and predictive analytics, AI can be used to optimize treatment plans for patients receiving deep brain stimulation (DBS). By analyzing patient data in real time, AI systems can adjust stimulation parameters to provide more precise therapeutic outcomes (21). This capability allows for continuous, personalized care, reducing the need for frequent hospital visits and improving the quality of life for patients with chronic movement disorders.

Another area where AI could expand its application is in mental health and cognitive disorders. AI-driven models have been developed to assist in the diagnosis of psychiatric and cognitive disorders like depression, schizophrenia, and mild cognitive impairment (MCI) (7). AI can analyze speech patterns, facial expressions, and behavioral data to detect early signs of cognitive decline, offering potential for early interventions in conditions like dementia. This approach provides clinicians with real-time insights that go beyond traditional cognitive assessments, allowing for a more dynamic and individualized understanding of patient progress.

Furthermore, AI can be instrumental in identifying biomarkers and developing predictive models for rarer conditions, such as multiple system atrophy (MSA) and progressive supranuclear palsy (PSP). These diseases often have overlapping symptoms with more common conditions, making early and accurate diagnosis challenging. AI algorithms trained on multimodal data—encompassing neuroimaging, clinical records, and genetic information—can help differentiate between these disorders, providing more timely and accurate diagnoses (33).

Despite these promising developments, there is still limited research on AI’s application to these broader neurological conditions. Future research must focus on expanding AI models to cover a wider array of neurological diseases, ensuring that these technologies benefit all patients, not just those with more commonly studied disorders. Collaborative efforts between neurologists, AI researchers, and healthcare providers will be essential in developing AI systems that can be generalized across diverse neurological conditions, leading to more inclusive and equitable healthcare solutions.

## 8 Comparative analysis: AI vs. traditional methods in neurological emergencies

For AI to truly transform the field of neurology, its applications must be critically compared to traditional diagnostic and treatment methods. While AI offers significant advantages in speed, precision, and the ability to process vast datasets, traditional methods still hold value in various clinical contexts, especially when considering the expertise of seasoned clinicians and the established reliability of manual diagnostic techniques.

One of the most well-recognized benefits of AI is its ability to analyze complex imaging data far more rapidly than human clinicians. For example, AI algorithms applied to CT and MRI scans in stroke cases can reduce diagnosis time from hours to minutes, a critical factor in improving patient outcomes (18). These algorithms can also detect subtle abnormalities that might be missed by human eyes, particularly in time-sensitive conditions like ischemic stroke, where quick identification of occlusions can determine whether a patient is a candidate for thrombolytic therapy (12).

However, traditional methods, particularly manual interpretation of imaging data, still play an essential role, especially in cases where clinical judgment and experience are needed to contextualize findings. For instance, while AI can flag potential abnormalities, it may struggle with atypical presentations that experienced radiologists or neurologists could interpret within the broader clinical picture (3). The complexity of neurological emergencies often requires the integration of multiple diagnostic inputs, including clinical history, laboratory results, and imaging studies—areas where human clinicians excel in synthesizing information for comprehensive decision-making.

Moreover, the robustness of AI in real-world clinical settings, particularly in low-resource environments, remains a significant concern. Traditional methods, though time-consuming, do not rely on advanced infrastructure, making them more accessible in healthcare settings with limited resources. This is a critical advantage when deploying diagnostic tools in rural or underserved areas, where AI-driven systems requiring high computational power and sophisticated hardware may not be feasible (34). In such settings, traditional methods remain the gold standard for ensuring patients receive adequate care.

Another area of comparison is in treatment personalization. AI-driven treatment plans, particularly for conditions like epilepsy and Parkinson’s disease, leverage vast amounts of patient data to recommend highly personalized therapies (9). However, traditional clinical decision-making, informed by years of medical experience, offers a level of intuition that AI systems currently lack. For example, while AI might suggest optimal DBS (Deep Brain Stimulation) settings for a Parkinson’s patient based on data, a neurologist’s familiarity with the patient’s nuanced responses to treatment can guide adjustments that enhance the patient’s quality of life (21).

Finally, AI and traditional methods differ in how they handle the element of uncertainty in neurological care. AI systems rely on probabilistic models that calculate the likelihood of outcomes based on available data. While this approach offers a level of precision and objectivity, it may fall short in cases where the data is incomplete or ambiguous. On the other hand, experienced clinicians are often adept at navigating uncertainty, making judgment calls based on intuition and years of practice—an area where AI has yet to match human capability (35).

In conclusion, AI offers clear advantages in terms of speed and data processing but still requires integration with traditional clinical methods, particularly in complex or resource-limited settings. Future research should focus on developing hybrid models that combine AI’s computational power with the clinical expertise of human neurologists, ensuring that both tools are used in complementary ways to improve patient outcomes in neurological emergencies.

## 9 Future directions and recommendations

### 9.1 Emerging trends in AI-assisted neurology

AI in neurology heralds a bright future with the lightning advances made in machine learning, data analytics, and computational power. Many emerging trends are set to shape the future of AI-assisted diagnosis and treatment of neurological disorders.

One of these trends is in multimodal approaches from AI frameworks, which combine different realms of data—imaging, genetic information, and clinical biomarkers—toward characterizing a condition for more complete and accurate diagnoses. Such systems combined from various sources can therefore help to realize much better diagnosis accuracy and avert several misdiagnoses that might be averted in hard neurological conditions. For example, genomics data can be combined with imaging results in diseases like brain tumors and neurodegenerative diseases to give valuable evidence for precision treatment strategies (24).

Neurogenomics represents a second most impactful emerging area that is being exposed by AI. Recent advances in the field of genomics and increasing volumes of available genetic data open up opportunities for AI discovery and corresponding targeted neurogenomic therapeutic interventions. Analysis driven by AI of these genetic data may well lead to more targeted therapy in general with respect to more accurate diagnoses of treatment—especially for serious genetically based conditions like Huntington’s disease and ALS (9).

In the case of another line, AI is growingly used to develop predictive models for the neurological disorder of interest. Such high-risk groups can lead, for example, to conditions such as Alzheimer’s or stroke, and early intervention and preventive therapy can be taken. Predictive analytics, with the help of AI, changes the field of neurology by switching it from a reactive to a proactive regime. It can avert the onset and reduce the progression of disease.

Another ray of hope is the integration of AI along with wearable devices and telemedicine platforms. Wearable devices with built-in AI models may offer continuous neuro health monitoring of patients, with the detection of initial manifestations of decline and real-time alerting to a healthcare provider. The importance of continuous monitoring cannot be overemphasized, since timely changes in treatment planning may prevent complications for such patients with chronic disorders as Parkinson’s disease or epilepsy.

### 9.2 Recommendations for clinical practice

Several catalyzers realize the full potential that AI can make in neurology for successful integration into clinical practice. Therefore, the following recommendations are proposed for healthcare providers along with policymakers and researchers to facilitate the adoption of AI technologies in neurology:

1. Infrastructure Investment in AI: Investment in the infrastructure needed, within healthcare institutions, to support AI technologies, such as advanced computing systems, data storage solutions, and secure networks. Such an investment is important in embedding AI systems into the existing workflow in the clinics in such a manner that it is best used.
2. IMPROVE QUALITY OF DATA AND DIVERSITY: AI models ought to be trained on diverse good quality data so that it can reduce bias and improve generalizability. Potential ways to ensure data are drawn from diverse patient populations, possibly including underrepresented groups, to ensure that AI systems are continuously oriented to operate equally across different subgroups, should an effort become prevalent toward these ends.
3. Promoting a Collaborative Interdisciplinary Field the Development of: Developing and applying AI in neurology requires a coordinated effort of AI development professionals, neurologists, and radiologists. It needs the cooperation of geneticists and other professionals working in healthcare. Systems developed by interdisciplinary teams will link the differences between technology and clinical practice into systems that are developed holistically to account for differences in the needs and priorities of patients and clinicians (30).
4. Ethical and Regulatory Compliance: AI developers should, therefore, put serious first consideration on ethics and observe regulatory standards. This pits several aspects that touch on privacy and data protection, informed consent issues, and general issues of accountability when AI systems are deployed into clinical settings, as has been postulated by numerous authors (29).
5. Investment in Clinician Education and Training: Both medical education curricula and training programs should include AI-augmented activities and protocols, preparing healthcare providers in this environment. Equally essential is having the knowledge clinicians must possess for better interpretation and integration of AI-sourced insights while providing care and recognizing AI’s capabilities and limitations in the process (36).
6. Support Ongoing Research and Development: Research in the development of AI applications for neurology needs continued support in order to either optimize existing solutions or keep pace with the development of new solutions. Specifically, funding agencies and academic institutions should ensure continuous support for research work that focuses on developing new AI solutions promising to support improvements in the diagnosis, management, and treatment of neurological disorders.

### 9.3 Research gaps and future studies

Despite its tremendous potential in the field of neurology, there are still certain important research gaps that need to be addressed in future studies. Among the most crucial, though, is another call for studies to be longitudinal investigations that assess the effects of AI-driven interventions over a long period of patient outcomes. Although most of the studies indicate the short-term benefits that AI brings, knowledge about its effects for a long term is of importance in knowing the worth or value conferred in clinical practice.

Another research gap is the development of explainable models of AI. Whereas AI systems could provide, with a good degree of accuracy, predictions and recommendations, they often acted like “black boxes” that are hard to understand at a deep level by clinicians. Development of transparent and explainable AI models will go a long way in engendering trust among healthcare providers in AI-driven decisions and ensuring that they are well understood and actionable (13).

In addition, ethical implications around issues of bias, fairness, and accountability necessitate more research on applied AI to neurology. It is a necessity to find ways in which biases in the AI systems are reduced to assure the developed technologies will be used equitably across all patient populations (37).

Future research in AI for neurological emergencies must focus on creating more interpretable and transparent AI systems. While deep learning models have shown great promise, their complexity often makes it difficult for clinicians to understand the rationale behind their recommendations. Developing explainable AI models will not only foster greater trust in these systems but also improve clinical adoption. Additionally, there is a need for longitudinal studies that assess the long-term outcomes of AI-driven interventions. Most current studies focus on immediate improvements, but the sustainability of these benefits over time remains unclear. Addressing these gaps is critical to ensuring the widespread and equitable application of AI in neurology.

Despite the promising strides made in AI applications for neurological emergencies, significant gaps remain in the current body of research. One key area that has been underexplored is the deployment of AI in low-resource settings. Most AI technologies in neurology are developed and tested in high-resource environments equipped with state-of-the-art imaging devices and computational infrastructure (34). However, in low- and middle-income countries (LMICs), where healthcare resources are scarce, the implementation of these AI systems faces considerable challenges. Research is urgently needed to modify AI systems for use in such settings, focusing on technologies that require less computational power, can work with limited or lower-quality data, and are cost-effective (38). The lack of studies in this area exacerbates the global inequality in healthcare access, and addressing this gap is crucial for ensuring that AI can benefit patients worldwide, regardless of geographic or economic barriers (43).

Another gap in the literature pertains to the long-term efficacy of AI-driven interventions. While there is substantial evidence showing the short-term benefits of AI in acute neurological conditions, such as rapid diagnosis and treatment of stroke (12), the impact of these interventions over extended periods remains unclear. For example, the use of AI in predicting stroke outcomes has demonstrated immediate improvements in treatment speed, but studies rarely follow patients long enough to evaluate the sustainability of these outcomes (18). Longitudinal studies are necessary to assess whether AI-driven improvements in the acute phase translate into long-term health benefits, particularly in managing chronic conditions like epilepsy and traumatic brain injury (TBI). Furthermore, these studies should investigate how AI-driven diagnostics and treatments affect patients’ quality of life, rehabilitation outcomes, and overall healthcare costs in the long term (35).

Additionally, the literature is disproportionately focused on a handful of well-known neurological conditions, such as stroke, Alzheimer’s disease, and epilepsy, while rare and complex conditions remain largely unexplored (9). For instance, there is a scarcity of studies examining AI’s potential in diagnosing and managing conditions like amyotrophic lateral sclerosis (ALS) and Huntington’s disease, despite the fact that these diseases could greatly benefit from the pattern recognition and predictive capabilities of AI systems (7). Expanding research to include these less common but equally devastating neurological disorders is essential for creating a comprehensive understanding of AI’s role in neurology.

Lastly, researchers need to develop knowledge of how the technology might be scaled up and made more accessible in the future, in the understudied conditions of low-resource use. Although developed, AI had captured significant goodwill where high-resource conditions prevail, several issues underscore the need for technologists and developers to develop insights about how these technologies will be transported for use in low-resource-exploiting conditions, where advanced medical care afforded by high-resource usage is too expensive. This is important for developing AI solutions that are affordable and scalable and therefore would ensure benefits in neurology are made available to all patients, regardless of their origin or economic status.
